# Pelvic sentinel lymph nodes have minimal impact on survival in melanoma patients

**DOI:** 10.1093/bjsopen/zrab128

**Published:** 2021-12-14

**Authors:** Mikko Vuoristo, Timo Muhonen, Virve Koljonen, Susanna Juteau, Micaela Hernberg, Suvi Ilmonen, Tiina Jahkola

**Affiliations:** Department of Plastic Surgery, University of Helsinki and Helsinki University Hospital, Helsinki, Finland; Department of Oncology, University of Helsinki, Helsinki, Finland; Department of Plastic Surgery, University of Helsinki and Helsinki University Hospital, Helsinki, Finland; Department of Pathology, University of Helsinki and Helsinki University Hospital, Helsinki, Finland; Department of Oncology, University of Helsinki and Helsinki University Hospital, Helsinki, Finland; Department of Plastic Surgery, University of Helsinki and Helsinki University Hospital, Helsinki, Finland; Department of Plastic Surgery, University of Helsinki and Helsinki University Hospital, Helsinki, Finland

## Abstract

**Background:**

Lower limb or trunk melanoma often presents with femoral and pelvic sentinel lymph nodes (SLNs). The benefits of harvesting pelvic lymph nodes remain controversial. In this retrospective study, the frequency and predictors of pelvic SLNs (PSLNs), and the impact of PSLNs on survival and staging was investigated.

**Methods:**

Altogether 285 patients with cutaneous melanoma located in the lower limb or trunk underwent sentinel lymph node biopsy of the inguinal/iliac lymph node basin at Helsinki University Hospital from 2009–2013. Patient characteristics, detailed pathology reports and follow-up data were retrieved from hospital files. Subgroups of patients categorized by presence of PSLNs were compared for outcome parameters including progression-free survival, melanoma-specific survival and groin recurrence.

**Results:**

Superficial femoral/inguinal SLNs were present in all patients and 199 (69.8 per cent) also had PSLNs removed. Median number of SLNs per patient was five and median number of PSLNs was two. Sixty-three patients (22.1 per cent) had metastases in their SLNs and seven (2.5 per cent) had metastases in PSLNs. A single patient had metastases solely in PSLNs, while superficial SLNs remained negative. Harvesting PSLNs or the number of PSLNs retrieved had no impact on progression-free survival or overall survival. The removal of PSLNs did not affect the risk of postoperative seroma or lymphoedema. The only predictor of positive PSLNs was radioactivity count equal to or more than that of the hottest superficial SLNs.

**Conclusion:**

Pelvic SLNs have minimal clinical impact on the outcome of melanoma patients especially in cases with negative superficial femoral/inguinal SLNs. Removal of PSLNs should be considered when they are the most radioactive nodes or equal to the hottest superficial femoral/inguinal SLNs in lymphoscintigraphy or during surgery.

Preliminary results were presented in part at the International Sentinel Node Society Biennial Meeting, Tokyo, Japan, 11–13 October 2018.

## Teaser

There is no consensus regarding the management of sentinel lymph nodes located in the pelvis in melanoma patients. In this study, the outcomes of 285 melanoma patients who underwent sentinel lymph node biopsy of the inguinal/iliac lymph node basin were analysed. Pelvic sentinel lymph nodes had minimal impact on survival and staging of melanoma patients, and removal of pelvic sentinel lymph nodes may be considered when they are the most radioactive nodes in lymphoscintigraphy or during surgery.

## Introduction

Sentinel lymph node biopsy (SNB) is a standard treatment of melanoma patients with no clinically detected metastases^1^^,^^2^. Sentinel lymph node (SLN) status is the most accurate predictor of survival with clinically negative regional lymph nodes^1^^,^^3^. Melanoma patients with primary tumour in the lower limb or trunk may present with pelvic (iliac/obturator) sentinel lymph nodes (PSLNs), with a reported incidence of harvested PSLNs varying from 8–23 per cent^4–8^. Considerable variation exists between centres and recommendations regarding the retrieval of PSLNs. In the authors’ centre, PSLNs have been routinely harvested whenever they present with radiotracer uptake and radioactivity clearly higher than background.

Anatomy of the lower extremity lymphatic system differs between individuals and even between sides of the body^9^. In general, lymphatic drainage of the lower extremity usually runs through the inguinofemoral lymph nodes (superficial sentinel lymph nodes, SSLNs) to PSLNs^10^^,^^11^. However, some variations occur^12^^,^^13^. Also, drainage from the trunk may run directly to the pelvic nodes^5^. Despite rare cases with lymphatic drainage directly to PSLNs, the vast majority of pelvic nodes are considered second-tier nodes^4^.

For the past couple of decades, a paradigm that positive sentinel node(s) led routinely to completion lymph node dissection (CLND) was followed tightly. CLND is generally associated with considerable morbidity such as seroma, wound dehiscence, infection, nerve injuries and lymphoedema^14^^,^^15^. This is even more evident in CLND of the inguinal or pelvic areas where frequent complications are reported^16–18^. Thus, previous studies aimed at selecting patients who could be managed without CLND, for example based on their low SLN tumour burden^19–21^. The MSLT-II and DeCOG-SLT multicentre studies showed no survival benefit for CLND^22^^,^^23^. This has led to a change in treatment protocol, with follow-up preferred to CLND for most patients with positive SLNs. However, there is no consensus regarding management of PSLNs.

In this study, the aim was to examine the benefit of harvesting PSLNs and the clinical impact of PSLNs in melanoma patients. The main objectives were to identify the frequency and predictors of metastases in SLNs and PSLNs and the impact of PSLNs on long-term progression-free survival (PFS) and melanoma-specific survival (MSS).

## Methods

The Helsinki University Hospital institutional review board approved the study protocol.

A total of 285 cutaneous melanoma patients whose primary melanoma was located in the lower limb or trunk with no clinically detected metastases at the time of the diagnosis and who underwent inguinal and/or iliac SNB at Helsinki University Hospital between 1 January 2009 and 31 December 2013 were included. The criteria for SNB included primary melanoma tumour Breslow classification greater than or equal to 1 mm and/or ulceration or mitotic level greater than or equal to 1/mm^2^.

Computerized medical records were reviewed in detail and data collected for each patient included age, gender, lymphoscintigraphy report, date and result of SNB and CLND, date of recurrence and/or death, type of recurrence and surgery-related adverse events.

For the purposes of this study, a seroma was considered significant when it demanded aspiration via needle or more invasive procedures. Similarly, lymphoedema was reported when compression socks were indicated. Melanoma-specific characteristics included anatomical site, histological type, tumour thickness and presence of ulceration.

The patients were routinely followed for a minimum of 5 years. Thirty-one patients with positive SLNs were randomized to the MSLT-II trial and followed according to the MSLT-II study protocol^22^.

### Protocol for sentinel lymph node operation

Lymphoscintigraphy was performed on the day before surgery. Patients received Technetium-99m-labelled colloidal albumin (Nanocoll, GE Healthcare, Amersham, UK) 80 MBq in 0.2 ml injection intradermally into the primary tumour site on both sides of the excision scar and then proceeded to lymphoscintigraphy with static images at 30 min and 2 h from injection. The surgeon used a gamma-detecting probe intraoperatively for SLN detection and harvested all radioactive nodes until no focal residual activity remained. The surgeon decided individually the technique used to harvest pelvic lymph nodes, guided by lymphoscintigraphy and gamma probe. In most cases, a separate incision on the abdominal wall was made to retrieve deep pelvic lymph nodes. The radioactivity count of each sentinel node was recorded.

### Processing of sentinel lymph nodes and completion lymph node dissection specimens

The SLNs were sent for histopathological analysis. Nodes were embedded in paraffin and cut serially into 1 mm slices and stained with haematoxylin–eosin. Immunohistochemical staining was performed with melanoma-specific antigens S-100, Melan-A and HMB-45.

The CLND specimen was weighed, and half of each node was taken for histopathological analysis (haematoxylin–eosin). Immunohistochemistry was not used routinely. Metastases were recorded according to size in one dimension and according to the number of positive nodes of all nodes in the basin.

### Statistical analysis

The clinicopathological co-variables of patients with and without PSLNs, as well as patients with and without positive SLNs were compared using a chi-squared test for categorical variables and Mann–Whitney U test for continuous variables. A multivariable logistic regression model was applied to analyse predictors of PSLNs, positive SLNs and positive PSLNs.

Progression-free survival and melanoma-specific survival were calculated from the time of SNB until first recurrence or death from melanoma, respectively, and censored if no such events had occurred by the last follow-up. Univariable analyses of survival were performed using the Kaplan–Meier method and the log rank test. Co-variables showing statistical significance in univariable analysis or considered to be of clinical importance were evaluated in a multivariable Cox proportional hazards model. No violation of proportional hazards assumption was found.

To elucidate any impact of melanoma staging according to the 8^th^ edition of American Joint Committee on Cancer staging manual^1^, the TNM classification and stage grouping of patients with the staging based solely on harvested SSLNs, that is a situation where no PSLNs had been removed, were compared.

SPSS^®^ version 25 (IBM, Armonk, NY, USA) was used for statistical analysis. *P* < 0.050 was considered significant.

## Results

### Patients and follow-up

The median age of patients was 58 years and two-thirds were female. The primary melanoma tumour (PMT) was located in the lower extremity, in or below the thigh, in 78.9 per cent of cases.

The median follow-up was 6.1 years. Of 285 patients, 62 (21.8 per cent) had recurrence of disease. The type of recurrence was local in 28 (45.2 per cent), regional in 13 (21.0 per cent) and systemic in 21 cases (33.9 per cent) respectively.

### Sentinel lymph nodes

All 285 patients had at least superficial femoral/inguinal SLNs. The median number of removed sentinel nodes was five (range 1–16). In addition, 199 patients (69.8 per cent) also had PSLNs. The median number of PSLNs was three (range 1–7).

In 183 cases (92.0 per cent), the most radioactive node was superficial. *Table 1* presents the baseline characteristics of 285 patients stratified by presence of PSLNs and 199 patients with PSLNs stratified by presence of positive SLNs.

**Table 1:** Clinical and histopathological characteristics of 285 patients by presence of pelvic sentinel lymph nodes and 199 patients with pelvic sentinel lymph nodes by presence of positive sentinel lymph nodes

	All patients				Patients with PSLNs		
	All (*n* = 285)	No PSLNs (*n* = 86)	PSLNs (*n* = 199)	***P***	No SLN+ (*n* = 157)	SLN+ (*n* = 42)	*P*
**Age (years)**							
Mean	57	55	57	0.612	57	58	0.746
Median (range)	58 (20–90)	58 (20–86)	58 (20–90)		58 (21–90)	60 (20–88)	
**Gender**							
Male	93 (32.6)	32 (37.2)	61 (30.7)	0.279	52 (33.1)	9 (21.4)	0.144
Female	192 (67.4)	54 (62.8)	138 (69.3)		105 (66.9)	33 (78.6)	
**Location of primary tumour**							
Trunk, groin or buttock	60 (21.1)	28 (32.6)	32 (16.1)	<0.001	27 (17.2)	5 (11.9)	0.216
Thigh	89 (31.2)	33 (38.4)	56 (28.1)		44 (28.0)	12 (28.6)	
Leg or ankle	86 (30.2)	15 (17.4)	71 (35.7)		59 (37.6)	12 (28.6)	
Foot	50 (17.5)	10 (11.6)	40 (20.1)		27 (17.2)	13 (31.0)	
**Breslow thickness (mm)**							
Mean	2.2	1.9	2.4	0.045	1.9	4.0	<0.001
Median (range)	1.5 (0.4–11)	1.3 (0.4–8)	1.5 (0.5–11)		1.3 (0.5–11)	3.5 (0.6–11)	
**Ulceration**							
Yes	67 (23.5)	20 (23.3)	47 (23.6)	0.887	25 (15.9)	22 (52.4)	<0.001
No	208 (73.0)	64 (74.4)	144 (72.4)		124 (79.0)	20 (47.6)	
Unknown	10 (3.5)	2 (2.3)	8 (4.0)		8 (5.1)	–	
**Number of SLNs**							
Mean	5.1	2.7	6.2	<0.001	6.1	6.7	0.297
Median (range)	5 (1–16)	2 (1–8)	6 (2–16)		6 (2–15)	6 (2–16)	
**Number of pelvic SLNs**							
Mean	1.9	–	2.7		2.6	3.1	0.065
Median (range)	2 (0–7)	–	3 (1–7)		2 (1–7)	3 (1–7)	
**Most radioactive SLN**							
Superficial	269 (94.4)	–	183 (92.0)		144 (91.7)	39 (92.9)	0.810
Pelvic	16 (5.6)	–	16 (8.0)		13 (8.3)	3 (7.1)	
**Patients with positive SLNs**	63 (22.1)	21 (24.4)	42 (21.1)	0.536	–	42 (100.0)	
**Follow-up time (years), mean**	6	5.7	6.5		5.7	6.5	
**Recurrent disease**	62 (21.8)	18 (20.9)	44 (22.1)	0.308	19 (12.1)	25 (59.5)	<0.001
**Groin recurrence**	16 (5.6)	2 (2.3)	14 (7.0)	0.113	4 (2.5)	10 (23.8)	<0.001
**Vital status, alive**	225 (78.9)	72 (83.7)	153 (76.9)	0.194	131 (83.4)	22 (52.4)	<0.001

Values in parentheses are percentages unless indicated otherwise. SLN, sentinel lymph node, PSLN, pelvic sentinel lymph node, SLN+, positive sentinel lymph node.

### Metastatic sentinel lymph nodes

Of all patients, 63 (22.1 per cent) had metastases in one or more SLNs. Seven patients (2.5 per cent of all patients and 11.1 per cent of those with positive SLN) had positive PSLNs. A single patient had metastases solely in PSLNs, while superficial SLNs remained negative. *Table 2* presents the baseline characteristics of 63 patients with positive SLNs stratified by presence of pelvic SLNs and positive pelvic SLNs.

**Table 2:** Clinical and histopathological characteristics of 63 patients with positive sentinel lymph nodes by presence of pelvic SLNs and presence of positive pelvic SLNs

	All (*n* = 63)	No PSLNs (*n* = 21)	PSLNs (*n* = 42)	*P*	No PSLN+ (*n* = 56)	PSLN+ (*n* = 7)	*P*
**Age (years)**							
Mean	57	56	58	0.770	57	56	0.562
Median (range)	60 (20–88)	58 (20–81)	60 (20–88)		60 (20–84)	52 (37–88)	
**Gender**							
Male	16 (25.4)	7 (33.3)	9 (21.4)	0.306	15 (26.8)	1 (14.3)	0.470
Female	47 (74.6)	14 (66.7)	33 (78.6)		41 (73.2)	6 (85.7)	
**Location of primary tumour**							
Trunk, groin or buttock	12 (19.0)	7 (33.3)	5 (11.9)	0.108	11 (19.6)	1 (14.3)	0.180
Thigh	18 (28.6)	6 (28.6)	12 (28.6)		17 (30.4)	1 (14.3)	
Leg or ankle	18 (28.6)	6 (28.6)	12 (28.6)		17 (30.4)	1 (14.3)	
Foot	15 (23.8)	2 (9.5)	13 (31.0)		11 (19.6)	4 (57.1)	
**Breslow thickness (mm)**							
Mean	3.6	2.9	4.0	0.051	3.6	3.9	0.474
Median (range)	3.0 (0.6–11)	2.5 (1.1–8)	3.5 (0.6–11)		3.0 (0.6–11)	4.5 (1.4–6.3)	
**Ulceration**							
Yes	30 (47.6)	8 (38.1)	22 (52.4)	0.285	26 (46.4)	4 (57.1)	0.593
No	33 (52.4)	13 (61.9)	20 (47.6)		30 (53.6)	3 (42.9)	
**Number of SLNs**							
Mean	5.3	2.7	6.7	<0.001	5.1	7.1	0.036
Median (range)	5 (1–16)	2 (1–7)	6 (2–16)		4 (1–16)	8 (4–9)	
**Number of PSLNs**							
Mean	2.1	–	3.1		1.9	3.9	0.016
Median (range)	2 (0–7)	–	3 (1–7)		1.5 (0–7)	3 (2–7)	
**Number of SLN+**							
Mean	1.7	1.7	1.7	0.953	1.4	3.7	<0.001
Median (range)	1 (1–7)	1 (1–4)	1 (1–7)		1 (1–4)	3 (2–7)	
**Most radioactive SLN**							
Superficial	60 (95.2)	21 (100)	39 (92.9)		34 (97.1)	5 (71.4)	0.002
Pelvic	3 (4.8)	–	3 (7.1)		1 (2.9)	2 (28.6)	
**Completion lymph node dissection**							
Positive	4	2	2	0.379	3	1	0.442
Negative	35	10	25		31	4	
**Follow-up time (years), mean**	5.2	5.4	5.1	0.988	5.3	4.6	0.505
**Recurrent disease**	33 (52.4)	8 (38.1)	25 (59.5)	0.131	27 (48.2)	6 (85.7)	0.172
**Groin recurrence**	11 (17.5)	1 (4.8)	10 (23.8)	0.060	7 (12.5)	4 (57.1)	0.003
**Vital status, alive**	39 (61.9)	17 (80.6)	22 (52.4)	0.028	36 (64.3)	3 (42.9)	0.271

Values in parentheses are percentages unless indicated otherwise. SLN, sentinel lymph node; PSLN, pelvic sentinel lymph node; SLN+, positive sentinel lymph node; PSLN+, positive pelvic sentinel lymph node.

### Completion lymph node dissection

Of 63 patients with positive SLNs, 39 (61.9 per cent) underwent a subsequent CLND. The reasons for avoiding CLND were as follows: randomized to MSLT-II follow-up group (19 patients), patient refusal (4 patients) and contraindicated due to poor general health (1 patient). In four cases (10.3 per cent) metastatic lymph nodes were detected in the CLND specimen. The CLND was continued to the iliac/obturator area in four patients (10.3 per cent) with positive pelvic SLNs. In this group, the patients had no further positive nodes in the CLND specimen.

### Predictive factors for pelvic sentinel lymph nodes

The location of the PMT was predictive of pelvic SLNs. Of 136 patients with the PMT located below knee level, 111 (81.6 per cent) had PSLNs harvested and of 149 patients with the PMT located above knee level, 88 (59.1 per cent) had PSLNs removed respectively (*P* < 0.001). The mean number of harvested SLNs was 6.1 when PMT was located below knee level and 4.2 when PMT was located above knee level (*P* < 0.001). Similarly, the mean number of PSLNs was higher in patients with PMT below knee level: 2.5 *versus* 1.3 respectively (*P* < 0.001). Mean PMT thickness was 2.4 mm in patients who had PSLNs removed and 1.9 mm in patients with no PSLNs removed (*P* = 0.045). Other parameters, such as ulceration, were not predictive of pelvic SLNs.

### Predictive factors for positive sentinel lymph nodes

*Table 3* presents multivariable analyses of co-variables as predictors of positive SLNs and positive PSLNs. PMT thickness (*P* < 0.001) and ulceration (*P* = 0.025) were predictive of positive SLN. The number of harvested nodes (*P* = 0.580), the presence of PSLNs (*P* = 0.536) or the number of harvested pelvic nodes (*P* = 0.333) had no impact on SLN status.

**Table 3:** Multivariable analyses regarding predictors of positive sentinel lymph nodes and positive pelvic sentinel lymph nodes

Co-variable	Presence of positive SLN		Presence of positive PSLNs	
	Odds ratio	P	Odds ratio	P
**Age**	0.98 (0.96, 1.00)	0.032	0.96 (0.91, 1.01)	0.127
**Gender (male)**	0.70 (0.34, 1.42)	0.320	0.42 (0.05, 3.90)	0.443
**Breslow thickness**	1.57 (1.30, 1.90)	<0.001	1.29 (0.92, 1.82)	0.144
**Ulceration**	2.38 (1.12, 5.06)	0.025	2.88 (0.46, 17.9)	0.257
**Number of SLNs**	1.00 (0.89, 1.11)	0.936	1.12 (0.93, 1.54)	0.169
**Hottest node pelvic**	1.52 (0.36, 6.43)	0.570	0.12 (0.02, 0.87)	0.036

SLN, sentinel lymph node; PSLN, pelvic sentinel lymph node.

### Predictive factors for positive pelvic sentinel lymph nodes

Radioactivity count of the SLNs was a predictor of positive PSLNs; that is, when the radioactivity count of the PSLNs was equal to or more than that of the hottest superficial SLN, it was more likely to harbour metastasis (*Table 2*). In multivariable analysis, it was the only significant predictor of positive PSLNs (*Table 3*). The likelihood of positive PSLNs was greater when the PMT was located in the foot (*P* = 0.050). Interestingly, however, all 12 patients with PMT in their toe presented with PSLNs, but none of these patients had positive PSLNs. Age, PMT thickness, ulceration and number of SLNs were not predictive of positive PSLNs.

### Impact on staging according to American Joint Committee on Cancer manual

When comparing the TNM classification and stage grouping of the patients to the staging based solely on harvested SSLNs, for 283 of 285 patients the staging remained the same^1^. The N category would have changed in six out of seven patients who had positive PSLNs. The patient who had metastases only in PSLNs would have been upstaged from IIA to IIIB group. In addition, one patient would have been upstaged from IIIC to IIID group.

### Prognosticators for survival

**Fig. 1:**
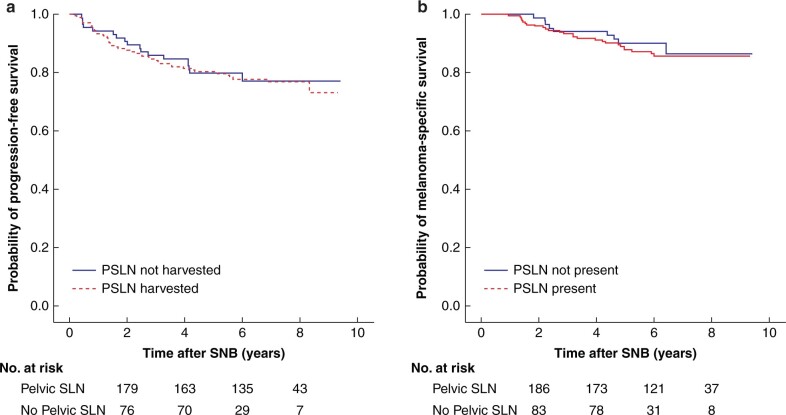
Kaplan–Meier plots of survival **a** Progression-free survival (hazard ratio 0.98, per cent confidence interval 0.57 to 1.68, *P* = 0.941) and **b** melanoma-specific survival (hazard ratio 1.20, 95 per cent confidence interval 0.56 to 2.56, *P* = 0.638) by presence of pelvic sentinel lymph nodes (PSLNs), whether harvested or not. SNB, sentinel lymph node biopsy.

In both univariable and multivariable analyses, age, PMT thickness, ulceration, PMT location in foot and positive SLNs were the strongest prognosticators for survival (*Tables 4* and *5*). The presence of PSLNs had no impact on PFS or MSS (*Fig. 1*). Also, the number of harvested pelvic nodes did not affect PFS or MSS (*P* = 0.436 and *P* = 0.251 respectively).

**Table 4:** Univariable analyses regarding survival of all patients

Co-variable	Progression-free survival		Melanoma-specific survival	
	Hazard ratio	*P*	Hazard ratio	*P*
**Age**	1.04 (1.03, 1.06)	<0.001	1.05 (1.03, 1.08)	<0.001
**Gender (male)**	0.80 (0.46, 1.38)	0.415	0.82 (0.39, 1.70)	0.591
**Location of primary tumour**				
Thigh Trunk, groin or buttock Leg or ankle Foot	1		1	
	1.46 (0.66, 3.19)	0.348	2.21 (0.70, 6.97)	0.175
	1.28 (0.61, 2.68)	0.521	2.21 (0.76, 6.46)	0.148
	3.85 (1.93, 7.67)	<0.001	5.56 (1.98, 15.61)	0.001
**Breslow thickness**	1.38 (1.28, 1.49)	<0.001	1.36 (1.23, 1.50)	<0.001
**Ulceration**	5.81 (3.53, 9.57)	<0.001	7.78 (3.87, 15.7)	<0.001
**Number of SLNs**	1.07 (0.98, 1.16)	0.128	1.09 (0.98, 1.22)	0.103
**Presence of PSLNs**	0.98 (0.57, 1.68)	0.941	1.20 (0.56, 2.56)	0.638
**Hottest node pelvic**	0.85 (0.31, 2.33)	0.747	2.16 (0.30, 15.8)	0.447

SLN, sentinel lymph node; PSLN, pelvic sentinel lymph node.

**Table 5:** Multivariable analyses regarding survival of all patients

Co-variable	Progression-free survival		Melanoma-specific survival	
	Hazard ratio	*P*	Hazard ratio	*P*
**Age**	1.02 (1.00, 1.04)	0.032	1.03 (1.00, 1.06)	0.023
**Gender (female)**	1.31 (0.71, 2.41)	0.388	1.23 (0.55, 2.74)	0.616
**Location of primary tumour**				
Thigh Trunk, groin or buttock Leg or ankle Foot	1		1	
	2.21 (0.90, 5.44)	0.084	3.64 (0.98, 13.4)	0.053
	1.84 (0.78, 4.35)	0.165	3.15 (0.95, 10.5)	0.061
	3.65 (1.52, 8.82)	0.004	4.37 (1.26, 15.2)	0.020
**Breslow thickness**	1.33 (1.18, 1.51)	<0.001	1.27 (1.07, 1.51)	0.006
**Ulceration**	2.58 (1.42, 4.68)	0.002	3.74 (1.65, 8.51)	0.002
**Number of SLNs**	0.98 (0.88, 1.08)	0.652	0.98 (0.86, 1.12)	0.792
**Presence of PSLNs**	1.43 (0.69, 3.00)	0.338	1.03 (0.38, 2.77)	0.955
**Hottest node pelvic**	0.64 (0.22, 1.88)	0.415	0.19 (0.03, 1.44)	0.108

SLN, sentinel lymph node; PSLN, pelvic sentinel lymph node.

**Fig. 2:**
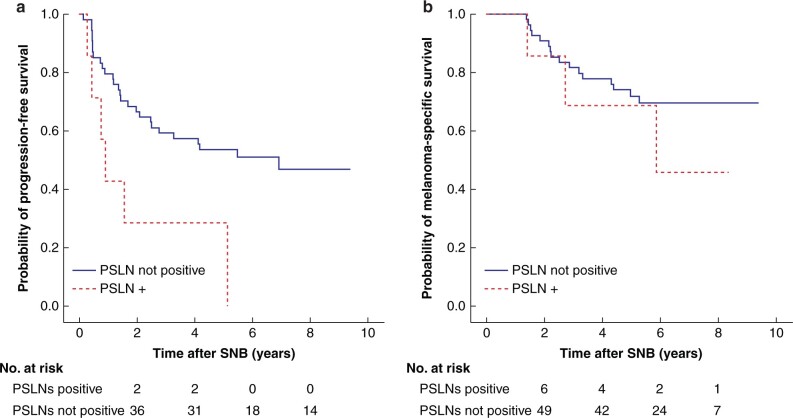
Kaplan–Meier plots of survival **a** Progression-free survival (hazard ratio 2.88, 95 per cent confidence interval 1.17 to 7.06, *P* = 0.016) and **b** melanoma-specific survival (hazard ratio 1.73, 95 per cent confidence interval 0.51 to 5.96, *P* = 0.376) by presence of positive pelvic sentinel lymph nodes (PSLN+) whether present or not. SNB, sentinel lymph node biopsy; PSLN, pelvic sentinel lymph node

In 63 patients with positive SLNs, patients with positive PSLNs showed a trend towards shorter PFS, but there was no difference in MSS (*Fig. 2*).

### Groin recurrence

Fourteen patients had a groin recurrence during follow-up, and four of them presented simultaneously with a systemic disease. Four patients with no SLN metastases had a groin recurrence, suggesting a false-negative rate of 6.0 per cent. Of 199 patients with PSLNs removed, 192 had no metastasis in PSLNs. Three of these 192 patients later presented with a pelvic recurrence. Two patients who underwent a superficial CLND developed metastases in pelvic nodes later in follow-up. PSLNs of six and three nodes, respectively, were negative in previous SNB for both patients.

### Seroma and lymphoedema

Of the overall study group, 74 patients (26.0 per cent), and 52 (21.1 per cent) of 246 patients who did not undergo CLND, presented with lymphoedema during follow-up. In multivariable analysis, female sex, location of PMT (foot) and total number of SLNs harvested were prognosticators for lymphoedema (data not shown). No difference was present between patients who had PSLNs removed and those who had not.

A seroma after SNB was present in 132 patients (46.3 per cent). No predictive co-variables were discovered, and again, no difference between groups emerged concerning the presence of PSLNs (data not shown).

## Discussion

The definition of a SLN is the first lymph node or nodes with direct lymphatic drainage from the primary tumour area^24^. The vast majority of pelvic lymph nodes are second-tier nodes^4^^,^^13^. In the current study, 69.8 per cent of patients had PSLNs removed in SNB with a median of three nodes. This represents a frequency far higher than has been published previously^4–8^. Yet only seven patients (11.1 per cent) with positive SLNs had positive PSLNs. Only one patient had positive pelvic nodes without any positive superficial SLNs in SNB. Interestingly, this patient had no metastatic nodes in either superficial or pelvic CLND specimens, but during follow-up developed a superficial groin metastasis.

Most importantly, there was no difference in melanoma-specific survival related to the presence of PSLNs in the overall study group or positive PSLNs among patients with positive SLNs. The harvesting procedure of pelvic SLNs adds to the operation time and causes additional surgical trauma and scarring. Therefore, other potential benefits are needed to justify routine retrieval of pelvic lymph nodes.

Positive PSLNs have been an indicator for CLND of iliac/obturator lymph nodes in addition to superficial lymph nodes^4^^,^^25–29^. After the results of MSLT-II and DeCOG-SLT, CLND has been omitted for the majority of patients with a positive SLN^2^^,^^22^^,^^23^. Thus, the extent of CLND becomes less important in most cases. New adjuvant therapies are promising and may well compensate whatever benefit CLND would theoretically provide for stage III patients^30–32^. Follow-up with ultrasonography and/or computed tomography is recommended instead of CLND. A therapeutic CLND may be advocated if nodal metastases are detected. Robot-assisted videoscopic surgery has recently gained popularity in pelvic lymph node dissection^33–35^.

On the other hand, a positive CLND specimen, with the presence of non-sentinel node metastases, represents a significant prognostic factor^22^. CLND status has been useful for staging and patient selection for clinical trials regarding adjuvant treatments^36^^,^^37^. Removing all potential SLNs would theoretically balance this missing information from CLND, and, therefore, removal of pelvic nodes would also be advocated. The current study does not support this hypothesis, as the number of harvested nodes was not predictive of either positive SLNs or survival. Furthermore, only two of 199 patients with PSLNs were upstaged based on their PSLN status. As the role of CLND has diminished, it is no longer among the inclusion criteria for most current adjuvant trials^38^.

In this study, the median number of harvested SLNs was five and the median number of harvested PSLNs was two per patient in the overall study group. SLNs were removed until no focal radioactivity remained rather than strictly following the widely used 10 per cent rule, that is harvesting SLNs with radioactivity of 10 per cent or more of that of the most radioactive node. The high numbers suggest that there may be more second-tier nodes removed than in other studies^4–8^. For the interests of this study, however, it was essential to remove all potential sentinel nodes in an attempt to identify any clinical impact. Despite the great number of harvested pelvic lymph nodes, they appear minimally important in the treatment and prognosis of melanoma patients.

In cases where the pelvic lymph node is the most radioactive or equal to the most radioactive superficial SLN, it is advisable to remove it. This may be difficult to determine intraoperatively, as the SLNs may be located deep in the pelvis. The role of lymphoscintigraphy must be highlighted when selecting true SLNs from the second-tier nodes. Dynamic imaging of sentinel nodes reveals potential direct pathways to pelvic lymph nodes, which indicates their removal.

A slightly shorter PFS was found in patients with positive PSLNs. Karakousis and colleagues observed a marginal association of presence of deep pelvic nodes with PFS in SLN-negative patients, suggesting that it may be a marker of more aggressive tumour biology^6^. In the current study, the presence of PSLNs was associated with higher Breslow thickness, supporting their theory.

Some limitations must be discussed in this retrospective study. There were differences between MSLT-II patients and other patients due to the randomization and follow-up protocol. Also, the data of complications, such as lymphoedema and seroma, were not collected in a prospective, standardized manner. However, they were routinely reported whenever present, and this study found no difference between patients with pelvic nodes harvested and those without. Other complications, such as chronic pain, were not investigated and might play a role when considering the drawbacks of harvesting PSLNs.

Since there is no survival benefit and the impact on staging is minimal, what remains to justify routine retrieval of PSLNs? Creating precise criteria for harvesting pelvic lymph nodes warrants randomized controlled trials or at least a large multicentre retrospective study, as suggested by Swords and colleagues^8^. Although only few studies exist on the importance of PSLNs, they mostly agree on the very limited impact of PSLNs on staging and treatment of PSLNs^6–8^. Apart from the rare cases where PSLN is the only or most radioactive SLN, it seems reasonable to omit routine retrieval of PSLNs.

PSLNs have minimal clinical impact on the outcome of melanoma patients, especially in cases with negative superficial femoral/inguinal SLNs. Removal of PSLNs should be considered when they are the most radioactive nodes or equal to the hottest superficial femoral/inguinal SLNs in preoperative lymphoscintigraphy or during surgery.

## Funding

M.V. was supported by a personal grant from the Vappu Uuspää Foundation. Apart from that, funding of this article was from departmental sources only. Open access was funded by Helsinki University Library. The funding source had no role in the design, execution, or publication of this study.
